# Late Presentation of Recurrent Solid Pseudopapillary Pancreatic Neoplasm With Liver Metastases During Pregnancy

**DOI:** 10.14309/crj.0000000000001418

**Published:** 2024-08-05

**Authors:** Ammad Javaid Chaudhary, Taher Jamali, Yara Dababneh, Abdulmalik Saleem, Reena Salgia

**Affiliations:** 1 Internal Medicine Department, Henry Ford Hospital, Detroit, MI; 2 Department of Gastroenterology and Hepatology, Henry Ford Hospital, Detroit, MI

**Keywords:** solid pseudopapillary epithelial neoplasms, SPEN, pancreatic neoplasms, pancreatic cancer, SPEN in pregnancy, SPN

## Abstract

Our case highlights a rare instance of recurrent metastatic solid pseudopapillary epithelial neoplasms of the pancreas, emerging 8 years after radical pancreatic resection—an extended interval surpassing the reported average. Managing solid pseudopapillary epithelial neoplasm during pregnancy is uniquely challenging, given the increase in the expression of progesterone receptors during the intrapartum period, leading to tumor growth. Although surgical resection remains the primary approach, systemic chemotherapy, radiation therapy, and liver transplant are other considerations. The absence of consensus guidelines for recurrence monitoring emphasizes the need for vigilant, long-term surveillance extending beyond the conventional 5-year mark.

## INTRODUCTION

Solid pseudopapillary epithelial neoplasms (SPENs) of the pancreas are rare, accounting for 1%–2% of all exocrine pancreatic tumors.^1^ These lesions are commonly seen in young females with a 10:1 predominance over males, most commonly in the second and third decades of life.^2,3^ Histopathologically, SPENs are usually well-circumscribed, with findings of necrosis, hemorrhage, and cystic degeneration. Although a thick, fibrous capsule is often present, a local desmoplastic reaction to the tumor is infrequent. On histology, the tumor comprises solid and pseudopapillary components characterized by uniform, polygonal epithelioid cells surrounding prominent microvasculature stalks.^3^

Most primary tumors (85%–90%) are localized to the pancreas at the time of diagnosis, with an increased prevalence in the tail of the pancreas. These tumors harbor low malignant potential with only 5% patients reported to develop metastasis.^4^ Although the standard-of-care treatment is surgical resection, there is no consensus or guidelines on SPEN management in pregnant patients. The only agreement on the matter is that it should be determined on a case-by-case basis. Recurrence is very rare after complete surgical resection.^3^ However, prognosis is generally favorable even in the setting of local recurrence or metastatic disease. The average time of disease after resection recurrence has been reported to be approximately 3.67 ± 2.42 years.^5^ In this article, we present a unique case of a significantly delayed presentation of SPEN recurrence many years after resection of the primary tumor and presenting symptomatically during pregnancy.

## CASE REPORT

**Figure 1.**
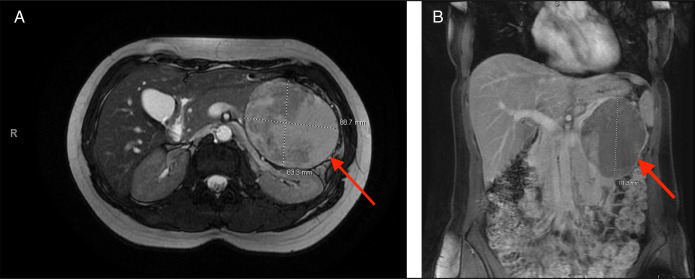
(A) Magnetic resonance imaging (transverse cross-sectional view) of original pancreatic solid pseudopapillary epithelial neoplasm (red arrow). (B) Magnetic resonance imaging (coronal cross-sectional view) of original pancreatic solid pseudopapillary epithelial neoplasm (red arrow)

**Figure 2.**
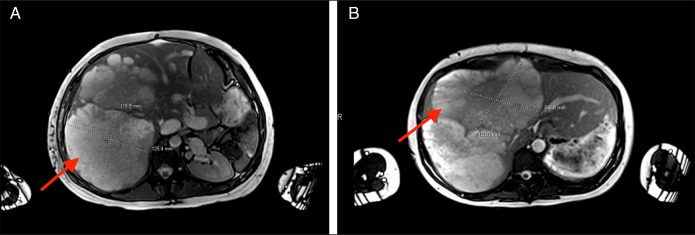
(A) Eight years after initial diagnosis and resection, magnetic resonance imaging (transverse cross-sectional view) of hepatic metastases of pancreatic SPEN (red arrow). (B) Eight years after initial diagnosis and resection, magnetic resonance imaging (transverse cross-sectional view) of hepatic metastases of pancreatic SPEN (red arrow).

A 28-year-old woman (G3 P2002) who presented with abdominal pain was found to have a sizeable pancreatic mass on imaging (Figure 1). She underwent a partial pancreatic and splenic resection. Pathology revealed pancreatic SPEN. A year later, surveillance imaging showed no evidence of recurrence, and she continued to do well. She gave birth to a healthy living baby 4 years after resection. No surveillance imaging was performed after the initial 1-year surveillance until 8 years later, when she presented with worsening abdominal pain, complicating her pregnancy at 22 weeks' gestation. Magnetic resonance imaging showed unexpected findings of multiple large complex cystic masses (largest mass: 14.9 × 12 × 17 cm) within the liver with associated hepatomegaly (Figure 2). A targeted liver biopsy confirmed a diagnosis of metastatic pancreatic SPEN. Staging imaging confirmed the absence of extrahepatic metastasis. Less than a month later, the patient was started on a gemcitabine regimen. She completed 2 cycles of therapy before delivering healthy twins at 34 weeks' gestation. Her liver metastases progressed, despite her chemotherapy. Because pf discomfort and the mass effect with vascular compression, she received 5 sessions of radiation therapy. Postpartum, she was started on FOLFIRINOX for continued systemic therapy.

**Figure 3.**
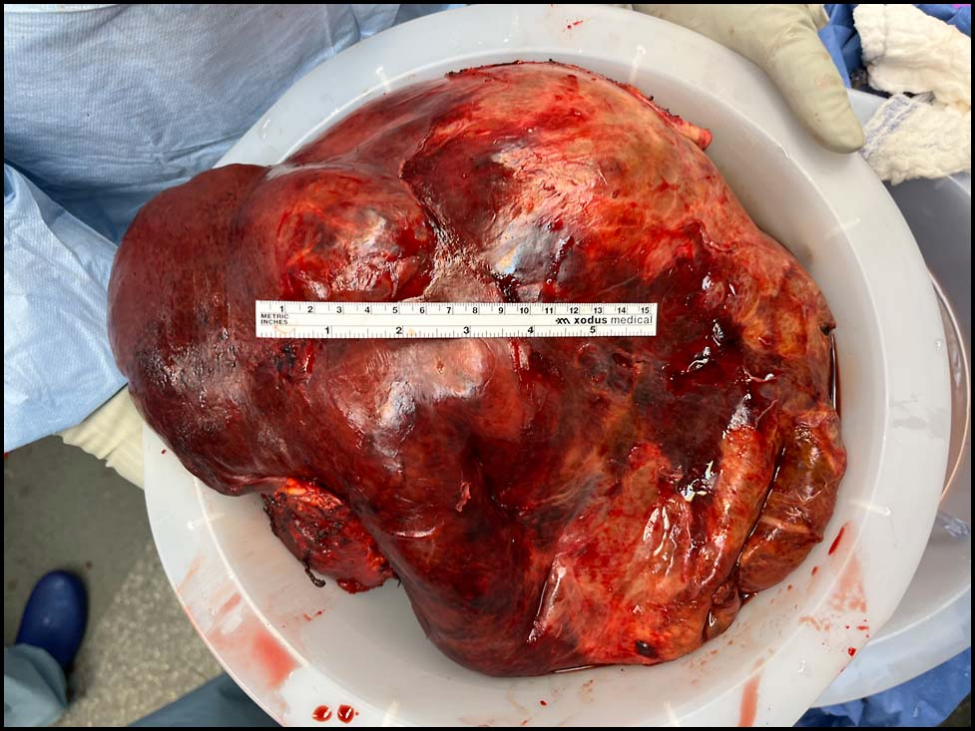
Gross sample of the tumor mass after a right liver trisegmentectomy with negative margins.

The patient developed portal hypertension with hepatic hydrothorax, ascites, and esophageal varices. In the setting of minimal residual hepatic reserve, a liver transplant workup was initiated (including a living donor evaluation because of a low Model for End-Stage Liver Disease score), and she was ultimately approved for listing. During that time, she was also evaluated for possible partial liver resection because it was believed that she had an adequate future liver remnant in the left lateral segment and multiple venous collaterals. She could still be listed for transplant if she developed acute decompensation postoperatively. She underwent a right liver trisegmentectomy with negative margins (Figure 3). Histology confirmed metastatic SPEN with extensive necrosis (comprising about 80%–90% of the gross tumor). She was discharged home on postoperative day 15.

## DISCUSSION

Our case presents a rare scenario of symptomatic metastatic SPEN diagnosed during pregnancy. The patient was managed initially with intrapartum and postpartum chemotherapy. She ultimately began developing decompensated liver disease from a significant tumor burden, which led to the consideration of surgical resection and/or liver transplant evaluation. The noteworthy feature of this case lies in the late recurrence of SPEN after 8 years, a substantial departure from the average recurrence time of approximately 3.67 years reported in the literature.^5^

SPEN management during pregnancy can be particularly challenging because progesterone receptors are often detected in these tumors, leading to accelerated growth and risk of rupture.^6^ Often times, Tanacan et al highlight the importance of monitoring intrapartum SPEN progression through abdominal ultrasound, emphasizing the need for increased vigilance in pregnant patients with a history of SPEN.^7^

Surgical resection remains the gold standard for SPEN treatment, with successful intrapartum surgical resection reported in numerous cases.^7–10^ However, because of an overall low incidence rate, there are no consensus guidelines for managing these lesions with chemotherapy and radiation.^10–12^ Maharaj et al report the successful use of neoadjuvant chemotherapy that allowed for further resection.^13^ Gemcitabine and FOLFIRINOX have also been used as neoadjuvant treatment, with FOLFIRINOX in nonpregnant individuals.^10,14,15^ In this case of pregnancy, management included intrapartum gemcitabine followed by postpartum FOLFIRINOX and radiation therapy.

Postresection recurrence, although uncommon, can increase patient mortality. Existing literature reports recurrence intervals ranging from 2 to 6 years.^16^ Most recurrences were noted within 5 years, with 1 study documenting a 6% recurrence after 84 months of follow-up.^10,17^ Some studies have also elucidated features that place individuals at higher risk of recurrence. Such features included tumor size >8 cm, synchronous metastasis, pancreatic parenchymal invasion, Ki-67 expression, stage IV disease, initial malignant disease, lymphovascular invasion, and R1 margins.^10,17^ Our patient's initial SPEN was >8 cm with pancreatic parenchymal invasion. However, the absence of R1 margins or lymphovascular invasion challenges the established risk factors for recurrence. Considering the lack of official guidelines for monitoring recurrence, our case underscores the need for vigilant, extended-term monitoring beyond the conventional 5-year mark or development of dynamic monitoring strategies based on risk assessment.^18^

Moreover, our case prompted consideration for a liver transplant because of the significant tumor burden in the liver and the lack of extrahepatic disease. Granat et al reported the first successful case of transplantation for SPEN metastasis, achieving remission on a 2-year follow-up.^19^ Given the low risk of recurrent disease and a favorable prognosis, transplantation from extended criteria donors may offer a viable option for this specific population or living donor liver transplantation.

In conclusion, our case contributes to the evolving landscape of SPEN management during pregnancy and challenges existing paradigms with its unique recurrence profile. The extended recurrence interval and successful management of marked hepatic metastases provide valuable insights. We recommend that all patients with SPEN have annual cross-sectional imaging surveillance for 10 years after resection with computed tomography or magnetic resonance imaging; however, magnetic resonance imaging should be considered when possible, given the generally young age of individuals (often in their third or fourth decade) with this condition. Moreover, we do not believe that a history of SPEN should trigger imaging before pregnancy based on the limited data at this time to support such a recommendation aside from annual follow-up for 10 years.

## DISCLOSURES

Author contributions: AJ Chaudhary: conceptualization of the case report, acquisition and interpretation of clinical data, and drafting and critical revision of the manuscript for important intellectual content; T. Jamali: substantial contributions to the diagnosis and treatment aspects of the case, review and interpretation of relevant medical literature, and critical revision of the manuscript for important intellectual content; Y. Dababneh: contribution to clinical management and patient care and involvement in the drafting and critical revision of the manuscript; A. Saleem: substantial contributions to the acquisition and interpretation of diagnostic data and critical revision of the manuscript for intellectual content; R. Salgia: oversight of the case report project, critical contributions to the conceptualization and design of the manuscript, substantial revisions and intellectual input during the manuscript preparation, and is the article guarantor.

Financial disclosure: None to report.

Previous presentation: Previously presented at the American College of Gastroenterology 2023 Annual Scientific Meeting; October 22, 2023; Vancouver, BC, Canada.

Informed consent could not be obtained for this case report. All identifying information has been removed.
